# The Apoptotic Role of Metacaspase in *Toxoplasma gondii*

**DOI:** 10.3389/fmicb.2015.01560

**Published:** 2016-01-19

**Authors:** Muzi Li, Hui Wang, Jing Liu, Pan Hao, Lei Ma, Qun Liu

**Affiliations:** Key Laboratory of Animal Epidemiology and Zoonosis, Ministry of Agriculture, National Animal Protozoa Laboratory, College of Veterinary Medicine, China Agricultural UniversityBeijing, China

**Keywords:** *T. gondii*, apoptosis, programmed cell death, metacaspase, knockout

## Abstract

*Toxoplasma gondii* is a major opportunistic pathogen that spreads in a range of animal species and human beings. Quite a few characterizations of apoptosis have been identified in *T. gondii* treated with apoptosis inducers, but the molecular mechanisms of the pathway are not clearly understood. Metacaspases are caspase-like cysteine proteases that can be found in plants, fungi, and protozoa in which caspases are absent. Metacaspases are multifunctional proteases involved in apoptosis-like cell death, insoluble protein aggregate clearance, and cell proliferation. To investigate whether *T. gondii* metacaspase (*Tg*MCA) is involved in the apoptosis of the parasites, we generated *Tg*MCA mutant strains. Western blot analysis indicated that the autoproteolytic processing of *Tg*MCA was the same as that for metacaspases of some other species. Indirect immunofluorescence assay (IFA) showed that *Tg*MCA was dispersed throughout the cytoplasm and relocated to the nucleus when the parasites were exposed to the extracellular environment, which indicated the execution of its function in the nucleus. The number of apoptosis parasites was significantly diminished in the *Tg*MCA knockout strain and increased in the *Tg*MCA overexpression strain after treatment with extracellular buffer, as determined by the terminal deoxynucleotidyl transferase dUTP nick end labeling (TUNEL) assay. The lack of *Tg*MCA did not affect the parasite propagation *in vitro* and virulence *in vivo*, suggesting that it is probably redundant in parasite propagation. But overexpression of *Tg*MCA reduced the intracellular parasites growth *in vitro*. The *Tg*MCA knockout strain showed more viability in extracellular buffer compared to the parental and overexpression lines. In this study, we demonstrated that *Tg*MCA contributes to the apoptosis of *T. gondii*.

## Introduction

Apoptosis, which is a form of programmed cell death (PCD), is important to the development and homeostasis of metazoans (Elomre, 2007). However, it remains controversial in protozoa because it leads to the death of a single-celled organism; how apoptosis might benefit a unicellular eukaryote is also difficult to understand (Reece et al., 2011). Recently, increasing evidence has demonstrated that regulated cell death occurs in single-celled organisms, although the dedicated molecular mechanism, and function remain unclear (Kaczanowski et al., 2011). To date, apoptosis has been described in *Trypanosoma, Leishmania, Giardia lamblia*, and *Plasmodium falciparum*, among other species (Bruchhaus et al., 2007; Gannavaram and Debranbant, 2012; Proto et al., 2013). Moreover, hallmarks of apoptosis in metazoans, including shrinkage and rounding of the body shape, externalization of phosphatidylserine (PS), DNA fragmentation and nucleus condensation, were observed and identified in the protozoa mentioned above, either naturally or under stimulation (Jiménez-Ruiz et al., 2010; Proto et al., 2013). Van Zandbergen et al. reported that *Leishmania* promastigotes contain a high ratio of apoptotic parasites that enable the intracellular survival of viable parasites in an altruistic way (van Zandbergen et al., 2006; Wanderley et al., 2013). The stumpy trypomastigote of *Trypanosoma brucei*, if it is not taken up by a tsetse fly for transmission, will undergo apoptosis in the mammalian host (Laverrière et al., 2012). *Toxoplasma gondii* appears to exhibit apoptotic characteristics when treated with H_2_O_2_, and the externalization of phosphatidylserine of parasites was identified in the peritoneal lavage fluids of mice infected with *T. gondii* by intraperitoneal injection (Santos et al., 2011; Nyoman and Lüder, 2013).

The mechanism of apoptosis in protozoa has not been studied in depth. Most of the genes that have been identified as participating in apoptotic pathways in metazoans are absent in the genome of parasitic protozoa. Hence, we hypothesized that the apoptosis pathway of protozoa may differ from that of mammals. Caspases are the central cysteine peptidases involved in apoptosis, but it is thought that protozoa might lack caspases. Metacaspases, orthologous to caspases, are found in plants, fungi, and protozoa in which caspases are absent (Uren et al., 2000). Metacaspases also belong to clan CD, C14 family, which exhibit significant structural similarity and a catalytic cysteine-histidine dyad compared with caspases (Carmona-Gutierrez et al., 2010). It is widely believed that the two types of metacaspases can be distinguished by the N-terminal prodomain, though another new one type of metacaspase has been found in phytoplankton (Choi and Berges, 2013). Type I metacaspases have an N-terminal prodomain, whereas type II metacaspases lack this prodomain but harbor a linkage between the p20 and p10 domains (Tsiatsiani et al., 2011). In protozoa and fungi, only type I metacaspases are found. More and more reports of functions of metacaspase are being published, and apoptosis has become a research focus. A cell death role for yeast Yca1 (the only metacaspase in Saccharomyces cerevisiae) was first demonstrated in budding yeast cell death (Madeo et al., 2002). Kosec et al. showed that TcMCA5 could be involved in the apoptosis of the parasites, as it was found to relocate from the cytoplasm to the nucleus during apoptosis induced by fresh human serum (Kosec et al., 2006). In Leishmania species, metacaspases were required for oxidative stress-induced cell death (Zalila et al., 2011). In the plant Norway spruce, metacaspase (mcII-Pa) causes developmental arrest at the early stage of embryogenesis by PCD (Suarez et al., 2004).

*T. gondii* is a major opportunistic pathogen that produces asymptomatic infection and cyst formation in the brains of normal people but can cause disease in immunosuppressed patients (Dubey, 2010). We conducted a comprehensive search in the *Toxoplasma* database (ToxoDB), and found three genes encoding metacaspase-like proteins in *T. gondii*. Here, we report the function of one of metacaspase in apoptosis and the phenotypic changes by the analysis of metacaspase null mutants.

## Materials and methods

### Animal approval statements

Our research with all animals were approved by the Beijing Laboratory Administration Committee in accordance with the recommendations in the Guide for the Care and Use of Laboratory Animals of the Ministry of Science and Technology of China (Approval No.: 18049).

### Parasites and cell cultures

Human foreskin fibroblast (HFF) and African Green Monkey kidney cell (Vero) lines were cultured in Dulbecco's Modified Eagle's Medium (DMEM) containing 25 mM glucose and 4 mM glutamine supplement with 10% fetal bovine serum (FBS, Gibco, USA), as previously described (Wang et al., 2014). *T*. *gondii* RHΔku80 (The Ku80 gene of the parasites had been deleted to improve the efficiency of gene targeting via double-crossover homologous recombination) tachyzoites (trophozoites, a stage of *T. gondii*, can infect almost any cell in the body and divides by endodyogeny) were maintained *in vitro* by serial passages on confluent Vero cells. Both cells and parasites were incubated at 37°C with 5% CO_2_ in a humidified incubator.

### Bioinformatics analysis of *Tg*MCA

The gene sequence and amino acid sequence of *Tg*MCA were obtained from ToxoDB (http://www.toxoDB.org, ver9.0). For further understanding, motif analysis (http://www.expasy.org/prosite), SMART (http://smart.embl-heidelberg.de/), and conserved domain predicition (CD research, http://www.ncbi.nlm.nih.gov) were used to obtain protein motifs and domains. The similarities to related proteins were analyzed by DNAman (Lynnon Biosoft, USA) and Mega5.0 (http://www.megasoftware.net). Sequence alignment analysis and three-dimensional structure modeling were performed using Espript3.0 (http://espript.ibcp.fr/ESPript/cgi-bin/ESPript.cgi) and Swiss model (http://www.swissmodel.expasy.org/).

### Cloning of *Tg*MCA and preparation of anti-r*Tg*MCA polyclonal antibody

Based on the gene sequence of *Tg*MCA, a PCR process was designed, but the complete coding sequence could not be obtained directly. Therefore, overlapping primers (Table S2) were designed to amplify the complete coding sequence, partly from RHΔku80 cDNA prepared from total RNA and partly from the RHΔku80 genome prepared using a DNA extraction kit (Aidlab Biotechnologies Co., Ltd., Beijing). Overlapping PCR was designed to obtain the complete coding sequence of *Tg*MCA using high-fidelity polymerase (Fastpfu, TansGenBiotech Co., Ltd., Beijing). The fragment was cloned into the pEASY-T blunt vector (TansGenBiotech Co., Ltd., Beijing), and sequencing result was correct. Then the complete coding sequence was cloned into the pET-28a vector (Novagen, Germany) and transformed into *Escherichia coli* (*E. coli*, Transetta, TansGenBiotech Co., Ltd., Beijing) for expression. The r*Tg*MCA was purified by affinity chromatography using Ni-IDA agarose (Novagen, Germany) following the Laboratory Manual of Molecular Cloning (Sambrook and Russell, 2000). The purified protein was assessed by SDS-PAGE.

Anti-TgMCA antibodies were obtained from 8 week-old female Balb/c mouse by immunizing 100 μg recombinant protein with the same volume of Freund's Adjuvant (Sigma, USA) per mouse. Anti-r*Tg*MCA serum was collected 2 weeks after the last immunization. The titers of polyclonal antibodies were examined by enzyme-linked immunosorbent assay (ELISA) using r*Tg*MCA as antigen. All serum samples were sterilized by filtration through 0.22 μm filters (Millipore, USA) and cryopreserved at −80°C.

### Western blot

Freshly isolated parasites were harvested and purified by filtration through a 5 μm filter, collected by centrifugation at 1400 × g for 10 min and washed in phosphate-buffer saline (PBS). Purified parasites were lysed with RIPA buffer (50 mM Tris pH7.4, 150 mM NaCl, 1% Triton X-100, 1% sodium deoxycholate, 0.1% SDS, Beyotime, Shanghai) with the protease inhibitor PMSF (Beyotime, Shanghai), and 7–10 μg of lysate was used for SDS-PAGE (12% w/v) electrophoresis and transferred onto polyvinylidene fluoride (PVDF) membranes (Millipore, USA). The membranes were blocked with 5% (w/v) skim milk in PBS. Then, membranes were incubated for 1 h at room temperature with mouse anti-r*Tg*MCA antibody or mouse anti-HA monoclone antibody (Santa Cruz Biotechnology, Inc., USA), diluted 1:500 in 5% skim milk in PBS and goat anti-mouse IgG(H+L) horseradish peroxidase (Sigma, USA) as secondary antibody, diluted 1:5000 in 5% skim milk in PBS, and incubated at room temperature. Finally, chemiluminescence reagents (CoWin Biotech Co., Ltd., Beijing) were used for reactive band visualization.

### Immunofluorescence assay

Immunofluorescence assays (IFA) for *Tg*MCA subcellular localization were performed as described previously (Wang et al., 2014). Appropriate amounts of parasites were seeded onto HFFs previously prepared on glass coverslips in 12-well plates. Infected cells were incubated at 37°C with 5% CO_2_ for no more than 20 h and fixed for 15 min in 4% formaldehyde, and then permeabilized with 0.25% Triton X-100 for 15 min and blocked with 3% bovine serum albumin (BSA) for 30 min. Subsequently, incubation was performed with mouse anti-r*Tg*MCA polyclonal antibody diluted 1:50 at 37°C for 1 h, then incubation was performed with FITC-conjugated goat-anti mouse IgG(H+L) (Sigma, USA) diluted 1:100 with 3% BSA for 1 h at 37°C. DNA of nucleus was stained with Hoechst33258 (Sigma, USA) for 5 min. The coverslips were observed and images were obtained using a Leica confocal microscope system (Leica, TCS SP52, Germany) with an oil immersion lens with 63 × magnification and the maximum emission wavelength of the fluorescent antibody (525 nm for FITC and 570 nm for cy3). Rabbit anti-*T. gondii* polyclonal antibody were used to stain the shape of the tachyzoites and pre-immune mouse serum were used as controls. The brightness and contrast of the images were adjusted using the LAS AF lite 2.2.0 software and the images were exported from this software. To analyze *Tg*MCA localization in extracellular parasites, filtered parasites were allowed to adhere to coverslips precoated with poly-lysine at 4°C overnight, and the IFA process and images collection were performed as above.

### Generation of *Tg*MCA knockout and overexpression strains

The parental strain used to generate the knockout strain was RHΔku80, which was transfected with the complete knockout plasmid PTCR-CD including genes of chloramphenicol and RFP (*T. gondii* positive selected marker gene) and the CD gene (bacterial cytosine deaminase, *T. gondii* negative selected marker gene). Briefly, ~2000 bp of the 5′ flanking and 3′ flanking sequence of *Tg*MCA were amplified from the RHΔku80 genome (primers in Table S3). The products were flanked with XhoI and HindIII, XmaI, and XbaI, respectively, and cloned into the PTCR-CD vector in order. The knockout plasmid was named PTCR-CD *Tg*MCA KO. PTCR-CD *Tg*MCA KO was used to disrupt the native loci of Δku80 tachyzoites by the double homologous recombination and replacement of the entire coding region, and stable clones were derived by chloramphenicol selection. Primers were designed to identify the correct clones (**Figure 3A**, Table S4), and finally the clones were confirmed by Western blot. For complementation analysis, the CRISPR/CAS9-UPRT specific sgRNA (kindly provided by Prof. BangShen, Shen et al., 2014) was used for the targeted disruption of the uracil phosphoribosyl transferase (UPRT) gene and used with resistance to fluorodeoxyribose (FUDR) for negative selection. Meanwhile, the *Tg*MCA complete coding sequences under the *Tg*GRA1 promoter flanked by UPRT 5′ and 3′ flanking sequences were co-transfected into *Tg*MCA knockout strain (Δ*Tg*MCA) and the parasites were selected by FUDR. To further characterize the role of *Tg*MCA, we generated a transgenic RHΔku80 strain overexpressing *Tg*MCA. The complete coding sequence of *Tg*MCA was amplified using primers that appended the flanking EcoRV and AvrII restriction endonuclease sites. Amplification products were introduced into the same site of a modified pDMG plasmid which GFP was replaced by HA, and the vector pDMG-*Tg*MCA-HA was electroporated into Δku80 for *Tg*MCA overexpression. The transgenic parasites were grown under selection pressure of pyrimethamine. The clones were identified by Western blot as described above.

### Plaque assay

The plaque assay was performed on HFF cells cultured in 6-well plates (Corning costar, USA) as described previously (Wang et al., 2014). A total of 500 freshly isolated parasites were seeded into HFF monolayers, and incubated at 37°C with 5% CO_2_ for 7 days undisturbed. After 7 days, the medium was removed from the HFFs, and they were washed 3–5 times with PBS. The cell monolayers were fixed with 4% formaldehyde for 10 min, stained with 0.2% crystal violet solution for 30 min and washed with deionized water, then visualized by microscopy (Olympus Co., Japan). The 6-well plates were scanned using an Canon digital scanner (Model: F917500, Japan). At least 50 plaques of each strain were chosen randomly and the plaque area was counted using Pixel in the Photoshop C6S software(Adobe, USA), and the data were compiled from three independent experiments. Statistical analysis was performed using One-way ANOVA with Tukey's *post-hoc* comparison. Differences were considered significant if the *P* ≤ 0.05.

### Intracellular parasite replication assay

Freshly isolated parasites (1 × 10^6^) were inoculated on HFFs in 12-wellplates (Corning costar, USA). After 30 min, the extracellular parasites were removed by washing 3–5 times with PBS and the plates were checked by microscope for the uninvaded parasites were removed completely. After incubation for 20 h, the infected cells were fixed with 4% formaldehyde, and the parasites were stained using rabbit anti-*T. gondii* positive serum following the IFA protocol. The number of parasites per vacuole were counted for each strain using a fluorescence microscope (Olympus Co., Japan) at 400 × magnification, and a total count of 100 parasitophorous vacuoles (PVs) of each strain was performed in each of three independent experiments. Statistical analysis was performed with the Chi-square test using statistical analysis system (SAS institute Inc., USA). Differences were considered significant if the *P* ≤ 0.05.

### Invasion assay

For the experimental testing of invasion, freshly isolated parasites were harvested, and 1 × 10^6^ parasites were seeded on HFFs in 12-well plates. After 30 min, the extracellular parasites were removed by washing for 3–5 times with PBS and the plates were checked by microscope for the uninvaded parasites were removed completely, then incubated for 24 h at 37°C with 5% CO_2_. To test the viability of extracellular parasites, parasites were pre-incubated in extracellular buffer (Ringer buffer, 155 mM NaCl, 3 mM KCl, 2 mM CaCl_2_, 1mM MgCl_2_, 3 mM NaH_2_PO_4_, 10 mM HEPES, and 10 mM glucose) for 1–2 h and seeded on HFF monolayers, and the extracellular parasites were removed and incubated as mentioned above. Subsequently, the medium was removed and fixed with 4% formaldehyde. To observe the parasite invasion and analyze the invasion ratio, IFA was performed following the method described above. Counting was performed using the fluorescence microscope, and data were compiled from three independent experiments, each in triplicate, counting eight randomly selected fields per clone at 400 × magnification. The ratio was calculated based on the number of infected cells divided by the number of total cells in one field of view. Statistical analysis was performed using Two-way ANOVA with Tukey's multiple comparison test. Differences were considered significant if the *P* ≤ 0.05.

### Virulence assay in mice

Virulence of the parasites were performed as described previously (Wang et al., 2014). Female, 8-week-old Balb/c mice were purchased from the Laboratory Animal Center of the Academy of Military. Rodent laboratory chow and tap water were provided, maintained under specific pathogen-free conditions and acclimatized for 7 days before each experiment. Parasites were injected intraperitoneally into the mice at doses of 10 or 100 tachyzoites (5 mice for each parasite strain), and mice were monitored until there were no survivors. All the infected mice were monitored every 8 h for clinical signs and mortality. The mice were humanely euthanized by the subcutaneous injection of atropine (0.02 mg/kg) or by cervical dislocation when they were unable to reach food or water for more than 24 h and lost 20% of normal body weight. The survival data were compiled from three independent experiment (dose of 100 tachyzoite) or two independent experiment (dose of 10 tachyzoites), 5 mice per stain in each experiment. Statistical analysis was performed using life test (life test data = surv) in statistical analysis system (SAS institute Inc., USA). Difference was considered significant if *p* ≤ 0.05.

### TUNEL assay

The terminal deoxynucleotidyl transferase-mediated dUPT nick end labeling (TUNEL) assay was performed as recommended by the manufacturer (Vazyme Biotech, Co., Ltd, Nanjing). Freshly isolated *T. gondii* was harvested by centrifugation at 1400 × g for 10 min, washed twice with PBS, and treated with Ringer buffer as a pro-stimulus for 1–6 h. Subsequently, the parasites were adhered to coverslips precoated with poly-lysine at 4°C overnight and fixed with 4% (w/v) paraformaldehyde in PBS for 15 min at room temperature. After the coverslips were washed, the parasites were permeabilized for 15 min with 0.1% Triton in PBS at room temperature. Then, the parasites were incubated in TUNEL reaction mix with TdT enzyme for 1 h at 37°C away from light. DNA of nucleus were stained with Hoechst33258 (Sigma, USA). To confirm the TUNEL-positive parasites, rabbit anti-*T. gondii* polyclonal antibody was used to stain the shape of parasites following the IFA protocol mentioned above and the samples which incubated with TUNEL reaction mix that did not contain TdT enzyme were used as control. The coverslips were observed, and images were obtained using a Leica confocal microscope system (Leica, TCS SP52, Germany). The number of TUNEL-positive parasites per image were counted at 630 × magnification, and 10 images of each strain were counted in each of three independent experiments. Statistically significant analysis was performed by One-way or Two-way ANOVA with Tukey's multiple comparison test. Differences were considered significant if the *P* ≤ 0.05.

### Statistical analysis

The statistical test of virulence assay and intracellular parasites replication assay were performed using statistical analysis system (SAS institute Inc., USA), and the other statistical test were performed in Statistical Product and Service Solution 22.0 (SPSS 22.0, IBM Co., USA). Data set were assumed to be equality of variance (determining by Homogeneity of variance in SPSS 22.0) and were analyzed with One-way analysis of ANOVA if only a single independent variable (e.g., genotype) or Two-way analysis of ANOVA if two independent variables (e.g., genotype and time). Tukey's *post-hoc* analysis was performed to comparing selected pairs of means. Two-way ANOVA was used to analyze parasites replication data, and using Chi-square for analysis each two parasite lines. In all cases, two-tailed *P*-value was calculated, and *P* ≤ 0.05 was consider significant.

## Results

### *Tg*MCA is a conserved cysteine-histidine catalytic dyad protein in *T. gondii*

**Figure 1:**
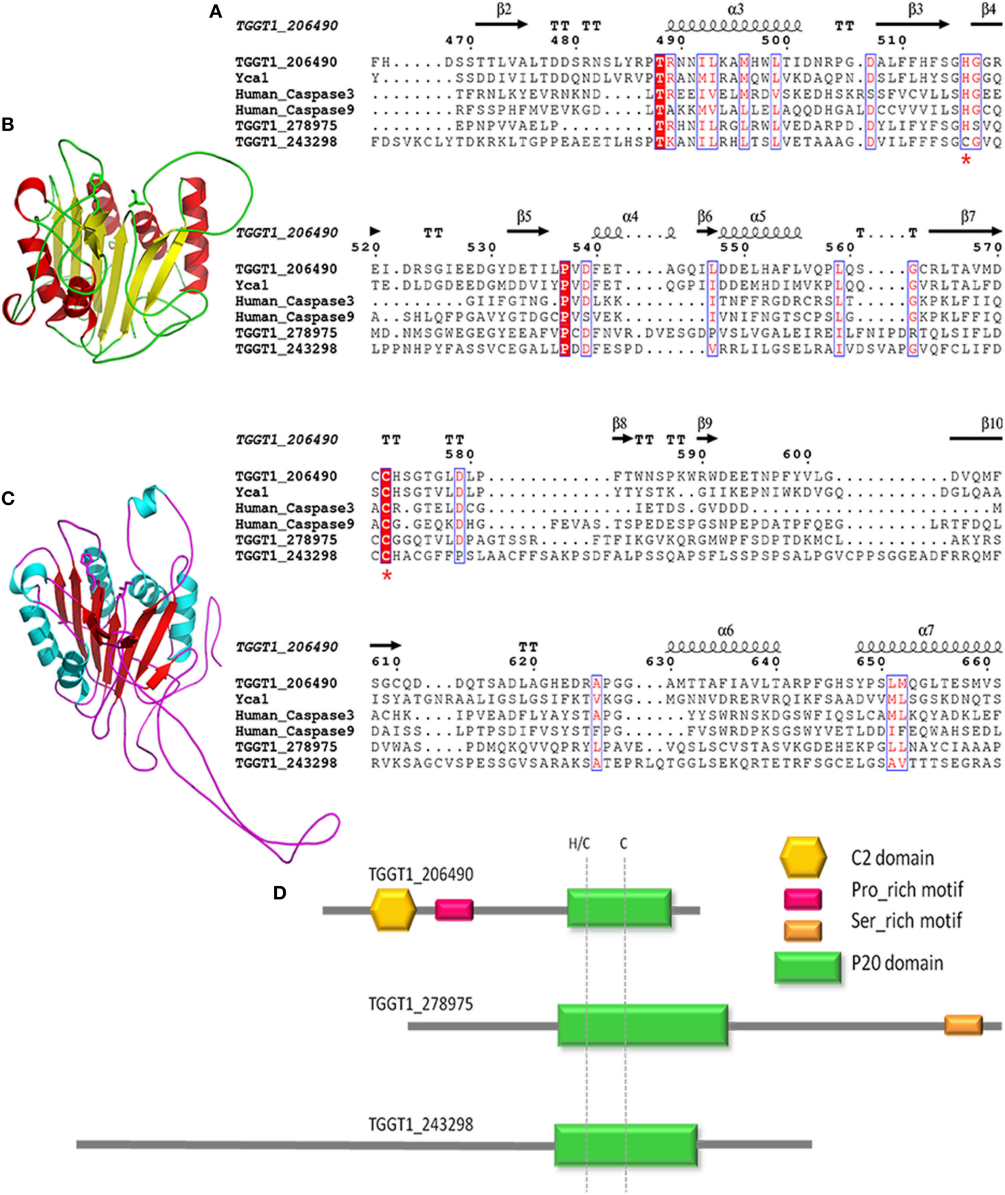
**Sequences-structure alignment of metacaspases. (A)** TGGT1_206490, TGGT1_278975, and TGGT1_243298 protein sequence were aligned with sequences of human Caspase3, Caspase9, and yeast Yca1. The positions of identical and conserved residues in p20 domain are indicated by red-filled and non-filled rectangular frames, respectively. The conserved histidine and cysteine are indicated with red asterisk. Dots indicate gaps or missing residues. **(B)** The ternary structure of *Tg*MCA was predicted by a model based on Yca1 **(C)** according to SWISS-Model and exhibited by Pymol. **(D)** Model pattern of the three ICE family proteins containing p20 domains in *T. gondii*, from top to bottom of the pattern, in order, TGGT1_206490, TGGT1_278975, and TGGT1_243298.

To obtain information on metacaspases in *T. gondii*, we used the *Toxoplasma* genomic resource database (ToxoDB, ver.9.0) to search for metacaspase-related genes. Proteins containing p20 domain were found in three genotypes in *T. gondii*, all of which belong to the ICE family protein based on the annotations (TGGT1_206490, TGGT1_278975, and TGGT1_243298). The three metacaspase genes are on different chromosomes of *T. gondii* and encode proteins sharing low amino acid identity because of the long and different N-terminal region (Table 1; Table S1). However, the three metacaspase proteins are definitely conserved in types I, II, and III *T. gondii* (different strains of *T. gondii*, based on the virulence to mice). We used the online service PROSITE for motif analysis and used SMART and CD research for conserved domain and protein structure prediction. Significant homology with the peptidase-C14/caspase domain was shown in the three metacaspases. A C2 domain (a Ca^2+^ dependent membrane targeting module), CUE domain (ubiquitin-binding CUE protein), and proline-rich motif were only present in TGGT1_206490, which were predicted to mediate protein-protein interaction (Figure 1D). Multiple alignment of the amino acid about the three metacaspases with Yca1 (the only metacaspase in *S. cerevisiae*, GI: 398365705), human caspase3 (GI: 704360691), and human caspase9 (GI: 17943347) was performed by ClustalW, and interestingly, only TGGT1_206490 carries histidine and cysteine as in yeast Yca1, which are predicted to form part of the catalytic dyad specific to clan CD. The conserved histidine or histidine adjacent to cysteine was absent from the other two metacaspases (Figure 1A, indicated by red asterisk). As previously reported, the mutation of cysteine and histidine in yeast Yca1 resulted in the complete abrogation of the catalytic process, and mutating the histidine adjacent to cysteine reduced the level of catalytic activity markedly (Wong et al., 2012). The ternary structure of full-length *Tg*MCA (Figure 1B) was modeled using the structural template of yeast Yca1 according to Swiss-Model (PDB: 4f6p.1.A, Figure 1C). *Tg*MCA encodes 742 amino acid, and 250 amino acid at C-terminal formed the p20 domain, which forming a middle β-sheet sandwiched by α-helices. The conserved cysteine and histidine residues were in the loops between β-sheet 3 and β-sheet 4 and between β-sheet 7 and β-sheet 8. Thus, in this study, we investigated the role of TGGT1_206490 (abbreviated to *Tg*MCA) in the apoptosis of *Toxoplasma* by constructing *Tg*MCA-deficient parasites.

**Table 1:** **Information on three different ICE family members containing p20 domain in ToxoDB**.

**Gene ID**	**Chromosome**	**Protein length**
TGGT1_206490	VIIa	742
TGGT1_278975	XII	1344
TGGT1_243298	VI	2055

### *Tg*MCA is expressed in tachyzoites

**Figure 2:**
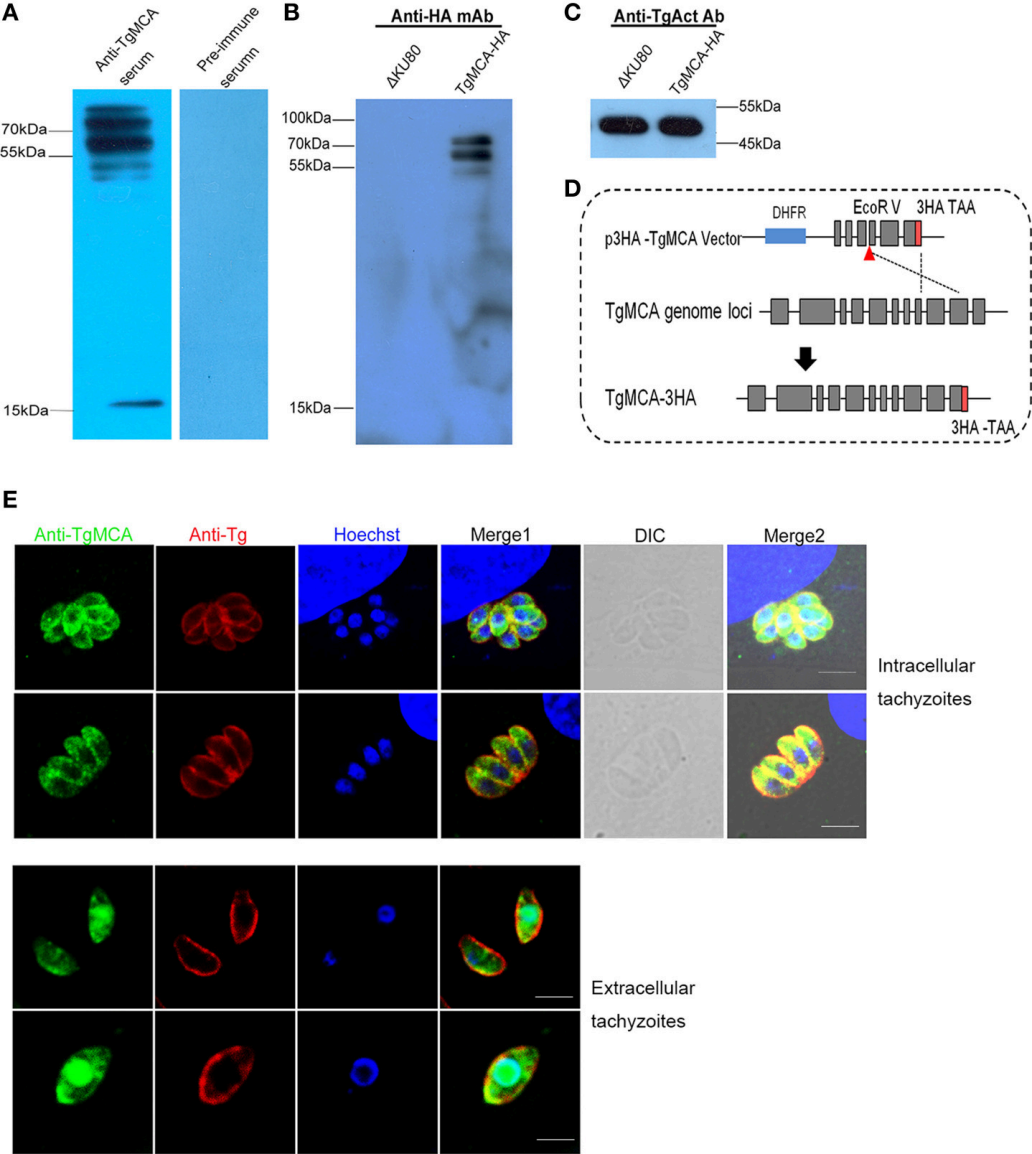
**Identification of ***Tg***MCA and cellular localization. (A)** Western blot analysis of native *Tg*MCA. Total antigens from cell cultured RHΔku80 tachyzoites. An expected band and two other bands were elicited by anti-r*Tg*MCA polyclonal antibody. Pre-immune serum was used as control. **(B)** Total antigens from transgenic tachyzoites with *Tg*MCA-HA and RHΔku80, two expected bands were revealed using anti-HA monoclonal antibody as primary antibody. **(C)** *Tg*Actin used as control for **(B)**. **(D)** Schematic of experimental design of *Tg*MCA endogenously marked HA. A knock-in vector was constructed to target *Tg*MCA endogenously marked HA at its C-terminal. **(E)** IFA analysis of *Tg*MCA localization. *Tg*MCA was distributed in the cytoplasm of the intracellular parasites and relocated to the nucleus of tachyzoites in the extracellular medium. The tachyzoites were stained with mouse anti-r*Tg*MCA serum (green) or rabbit anti-*T. gondii* serum (red, stain the shape of the parasites), and the nucleus DNA was stained with Hoechst (blue). Scale bar, 5 μm.

To assess the expression and the localization of *Tg*MCA in the tachyzoites, the full-length *Tg*MCA protein fused with a histidine-tag was successfully expressed in *E. coli* and used to generate anti-r*Tg*MCA serum in mice. The anti-r*Tg*MCA polyclonal antibody reacted with an 80 kDa protein in the lysate of tachyzoites as an antigen by western blot, and the pre-immune mouse serum exhibited no reactivity with the parasites (Figure 2A). More interestingly, two other molecular weights of positive protein bands, ~65 kDa and 15 kDa, were observed. Previous studies on metacaspases in some other species have demonstrated that metacaspases are autoproteolytically processed (Meslin et al., 2007; Wen et al., 2013). To confirm whether *Tg*MCA undergoes proteolytic processing, endogenous *Tg*MCA was fused with HA-tag through plic-HA vector knocking in at the C-terminal of the *Tg*MCA locus in *T. gondii* (Figure 2D). The lysate of the tachyzoites of which *Tg*MCA was marked with HA reacted with anti-HA monoclonal antibody using western blot. The results showed that an ~80 kDa band and a 70 kDa band reacted against the antigen (Figure 2B), and *Tg*Act was used as control (Figure 2C). Taken together, these results suggest that *Tg*MCA could be autoprocessed in tachyzoites.

Indirect immunofluorescence assay (IFA) analyzed the subcellular localization of *Tg*MCA in RHΔku80 using mouse anti-r*Tg*MCA serum (Figure 2E). *Tg*MCA localized in the cytoplasm of the intracellular parasites. However, interestingly, the *Tg*MCA of the extracellular parasites translocated into the nucleus and was concentrated in the nucleus of the parasites, co-localized with the nucleus DNA dye Hoechst. This results suggest execution of function of *Tg*MCA is in the nucleus of the extracellular parasites.

### *Tg*MCA knockout, complementary, and overexpression strains were successfully constructed

**Figure 3:**
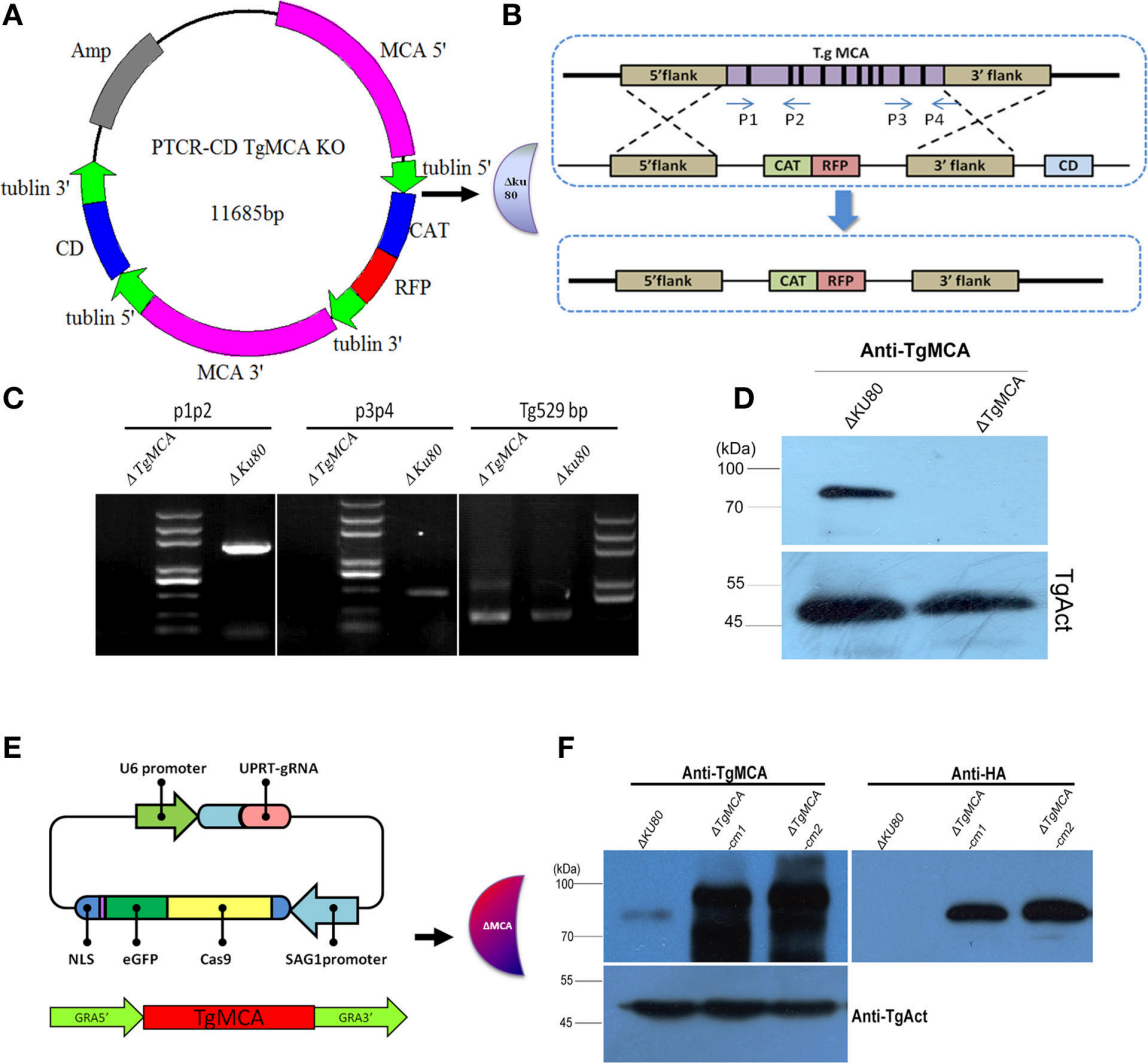
**Generation of a ***Tg***MCA mutant strain**. **(A,B)** Schematic of the experimental design of the *Tg*MCA knockout strain. A knockout vector (PTCR-CD *Tg*MCA KO) was constructed to target the *Tg*MCA complete gene. **(C)** Genomic PCR analysis of Δ*Tg*MCA strain. The position of the primers are shown in **(B)**. P1, P2 and P3, P4 were used to amplify exon2 to exon3 and exon10 to exon11 of *Tg*MCA, respectively. **(D)** Western blot performed with anti-r*Tg*MCA antibody on total extracts from Δku80 and Δ*Tg*MCA, with *Tg*Actin used as control. **(E)** Schematic of the complementary strain construction. Crispr/Cas9-UPRT was used to target the UPRT locus of Δ*Tg*MCA, and *Tg*MCA coding sequence fused with the HA-tag at the C-terminal under the GRA1 promoter was inserted into the gap of the UPRT locus. **(F)** Western blot analysis of the restoration of *Tg*MCA with anti-r*Tg*MCA serum and anti-HA antibody on total lysate of Δku80 and Δ*Tg*MCA-cm, with *Tg*Actin used as control.

To characterize the biological role of *Tg*MCA, we generated a complete knockout mutant of *Tg*MCA (Δ*Tg*MCA) by targeting the native *Tg*MCA locus in the RHΔku80 strain, using a homologous recombinant approach to replace the *Tg*MCA gene with a CAT-RFP cassette in the parasite genome (Figures 3A,B). Successfully targeted gene deletion was confirmed by PCR and western blot (Figures 3C,D, Table S4), and the parasites were resistance to chloramphenicol and the RFP was well distributed in the cytoplasm of the parasites. Western blot analysis showed no reactivity of Δ*Tg*MCA using antibodies against *Tg*MCA, with antibodies against *Tg*Actin as control. Re-expression of *Tg*MCA was accomplished by transfection into Δ*Tg*MCA with the Crispr/Cas9-UPRT vector and complete *Tg*MCA coding sequence bearing the HA-tag at the C-terminal under the GRA1 promoter (Figure 3E). The expression of *Tg*MCA was at high level in the complementary strain by western blot as the strong promoter (Figure 3F) and named Δ*Tg*MCA-cm. We also constructed a *Tg*MCA overexpression strain (*Tg*MCA OE), electroporated with modified pDMG-TgMCA into RHΔku80 (Figure S1A). After 10 generations of selection by pyrimethamine, a trangenic RHΔku8 stably expressing *Tg*MCA fused with the HA-tag was isolated by limiting dilution in 96-well plates and confirmed by western blot (Figure S1B). To ensure the role of *Tg*MCA in the overexpression strain was not affected by the plasmid pDMG-*Tg*MCA insertion in the genome of the parasites, two clones were selected for further investigation.

### *Tg*MCA is required for apoptosis response of tachyzoites

**Figure 4:**
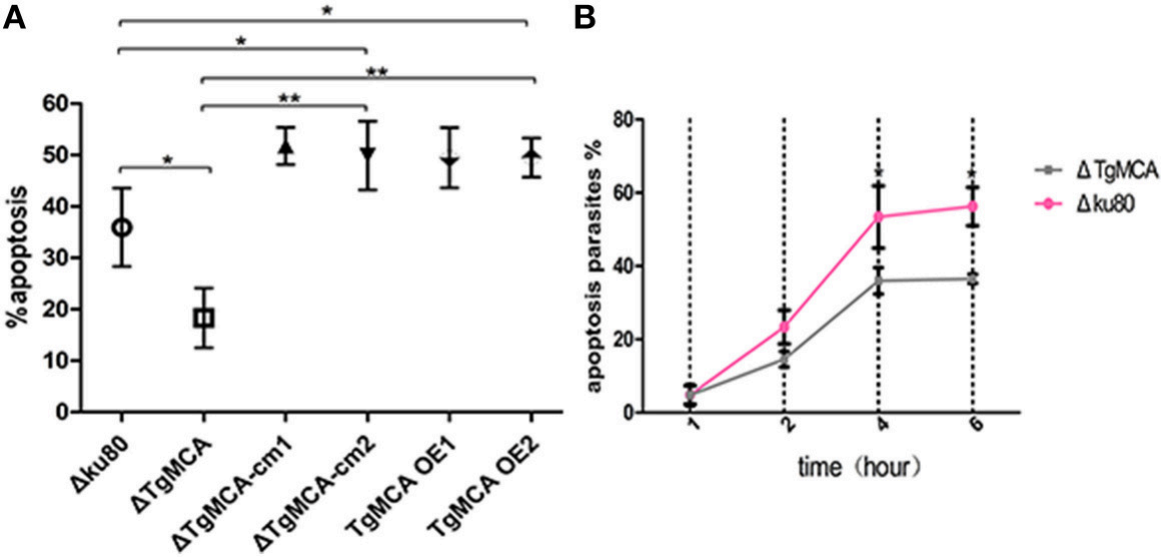
**Extracellular Δ***Tg***MCA is less sensitive to environmental stress. (A)** Ratio of apoptotic parasites by TUNEL assay. Apoptotic Δ*Tg*MCA was significantly decreased when treated with BAG buffer for 4 h (*p* ≤ 0.05). *Tg*MCA OE Δ*Tg*MCA-cm1 and Δ*Tg*MCA-cm2 produced far more TUNEL-positive parasites than Δku80 (*p* ≤ 0.05, *p* ≤ 0.01). Asterisks indicate statistically significant results as determined by One-way ANOVA with Tukey's *post-hoc* comparison. **(B)** Ratio of apoptotic parasites pre-incubated in Ringer buffer for 1–6 h by TUNEL assay. Both strains showed increased apoptotic ratio. Apoptotic Δku80 was higher than Δ*Tg*MCA after 2 h in extracellular buffer and significantly increased at 4 h (*p* ≤ 0.05). Asterisks indicate statistically significant results (*p* ≤ 0.05) as determined by Two-way ANOVA with Tukey's *post-hoc* comparison. Data are mean ± SD (error bars) of three independent experiment.

In our previous study, we observed that tachyzoites isolated from a host cell exposed to the extracellular environment would undergo apoptosis by the TUNEL assay, whereas intracellular parasites sheltered in the PVs would proliferate well and not become apoptotic without drug treatment (Nyoman and Lüder, 2013). To study the potential role of *Tg*MCA in the apoptosis pathway of the parasites, we used the TUNEL assay to quantify the apoptotic ratios of Δku80, Δ*Tg*MCA, Δ*Tg*MCA-cm, and *Tg*MCA OE strains treated with extracellular buffer for 4 h. We found that the number of TUNEL-positive Δ*Tg*MCA parasites was 17.93% significantly lower than that for Δku80, which is 33.83% after exposure to extracellular buffer (Figure 4A), and apoptosis of Δ*Tg*MCA-cm1 and Δ*Tg*MCA-cm2 parasites were increased with the high level expression of *Tg*MCA under GRA1 promoter (Figure 4A). The *Tg*MCA OE strains were sensitive to the extracellular environment, with TUNEL-positive parasites at 53.8 and 49.35% (Figure 4A). We also counted the number of apoptotic-response parasites in extracellular buffer during 1–6 h. Figure 4B shows that ~5% of both parasites were TUNEL positive (4.82% for Δku80 and 4.73% for Δ*Tg*MCA) for 1 h. Then the apoptotic ratio of the parasites was evaluated by ionic stimulation, but Δ*Tg*MCA still showed an obviously lower number of TUNEL-positive parasites, which indicates that *Tg*MCA participates in the apoptotic-response pathway. Meanwhile, the mean fluorescence intensity of the images were obtained by confocal microscopy and analyzed. The FITC-fluorescence intensity (TUNEL positive) of Δ*Tg*MCA was lower than for Δku80 (Figure S2A), which corroborated the results above.

The transcription level of the other two ICE family proteins were detected by real-time PCR. According to three independent real-time PCR experiment, no significant transcriptional difference in TGGT1_278975 and TGGT1_243298 occurred in the extracellular Δ*Tg*MCA compared to Δku80 (Figure S2B, primers in Table S5).

### *Tg*MCA knockout didn't influence the growth of parasites *in vitro* or virulence *in vivo*

**Figure 5:**
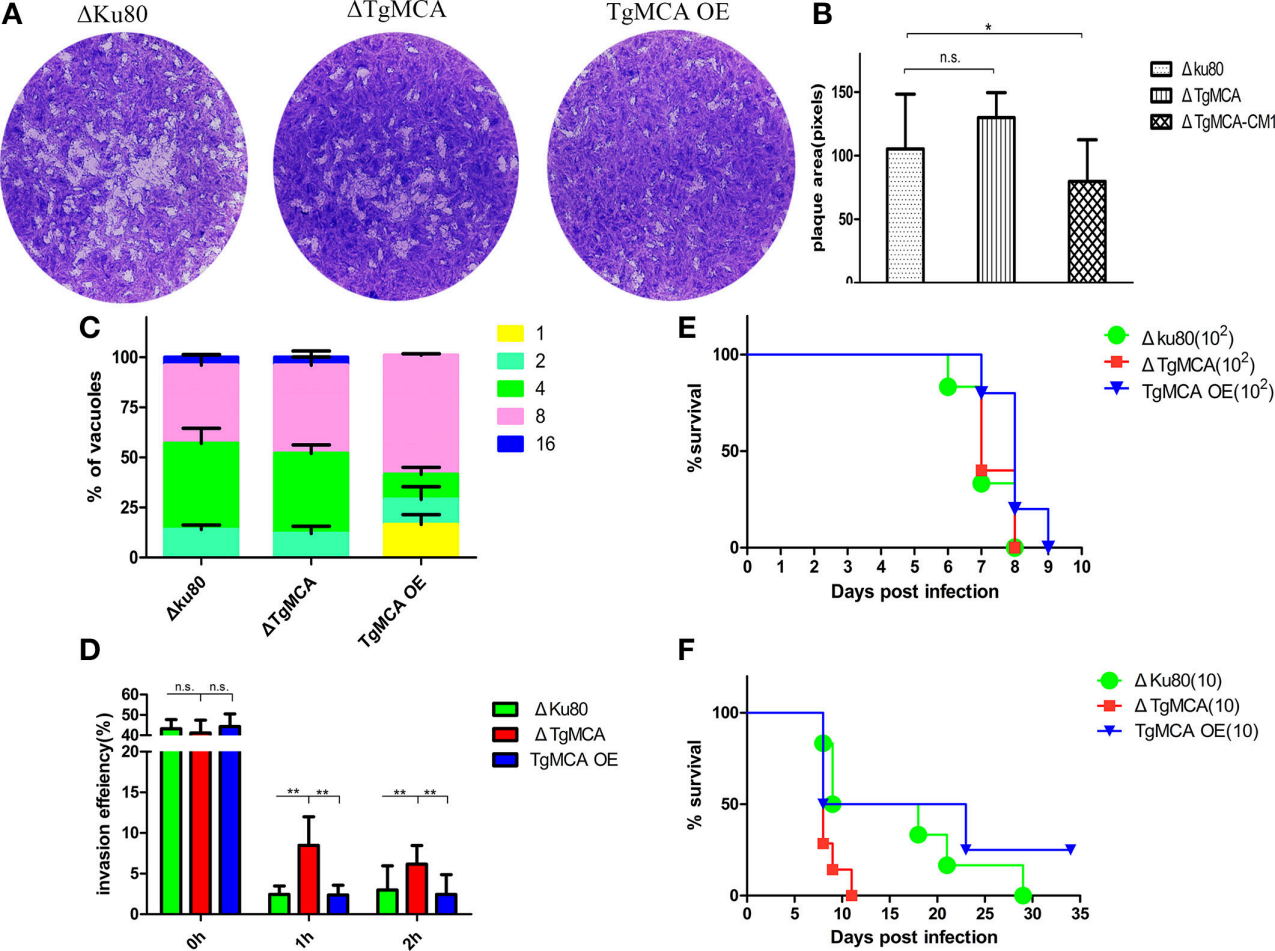
**Lack of***Tg***MCA did not affect intracellular parasite replication but did affect parasite viability in the extracellular environment. (A)** Plaque assay comparing growth of Δ*Tg*MCA, Δku80, and *Tg*MCA OE. Each well (HFF cell) was infected with 500 parasites, and plaques were stained 7 days later. Data were compiled from three independent assay. **(B)** The plaque areas were counting by randomly chosen at least 50 plaques and using the Pixel point in Photoshop C6S software (Adobe, USA), and the data were compiled from three independent experiment. Analysis of plaque area was performed using One-way ANOVA with Tukey's *post-hoc* comparison. Asterisks indicated significant results (*p* ≤ 0.05). **(C)** Intracellular parasite replication of Δ*Tg*MCA, Δku80, and *Tg*MCA OE. Data were compiled from three independent assay, and in each assay 100 total PVs of each strain were counted. Data were determined by Chi-square analysis. **(D)** Invasion ratio of Δ*Tg*MCA, Δku80, and *Tg*MCA OE, freshly isolated and pre-incubated in extracellular buffer for 1 or 2 h. The ratio was based on the number of cells infected with parasites divided by the number of total cells in one field of view. Asterisks indicated statistically significant results (*p* ≤ 0.05 as determined by Two-way ANOVA with Tukey's *post-hoc* comparison). Data are mean ± SD (error bars) of three independent experiment. **(E,F)** Mouse survival after infection with different doses of Δ*Tg*MCA, Δku80, and *Tg*MCA OE. Balb/c mouse were injected intraperitoneally (i.p.) with 100 or 10 indicated parasites. There were 5 female mice in each group, statistical analysis was performed using life test (life test data = surv) in statistical analysis system (SAS institute Inc., USA). The figures are representative of three experiments (a dose of 100 tachyzoites) and two experiments (a dose of 10 tachyzoites) with similar outcomes.

Because our results showed that *Tg*MCA was involved in the pathway of apoptosis of *T. gondii*, we therefore investigated the invasion, replication, and virulence of Δ*Tg*MCA and *Tg*MCA OE. The invasion ability of Δ*Tg*MCA, Δku80, and *Tg*MCA OE, all freshly isolated from Vero cells, were almost the same; however, after treatment with extracellular buffer for 1 h, Δ*Tg*MCA exhibited a stronger capacity of invasion than Δku80 and *Tg*MCA OE (Figure 5D). The lack of *Tg*MCA did not affect parasite growth *in vitro*, as the plaque area of Δ*Tg*MCA were equivalent to Δku80 (Figures 5A,B). The number of tachyzoites in each PVs of Δ*Tg*MCA and Δku80 were almost the same, which indicated *Tg*MCA knockout did not affect in the replication of the parasites. More interesting, overexpression of *Tg*MCA affected proliferation of the parasites, as the plaque area of *Tg*MCA OE was smaller than the other two strains and 16.5% PVs contained only 1 tachyzoites according to the replication assay (Figures 5A–C). The results above suggest that *Tg*MCA knockout strain show more viability after exposed to the extracellular buffer but did not affect the parasite growth and replication, and overexpression of *Tg*MCA affected the proliferation of the parasites *in vitro*.

Finally, we evaluated the contribution of *Tg*MCA to the virulence of parasites *in vivo*. We infected mice intraperitoneally with 10 tachyzoites or 100 tachyzoites of Δ*Tg*MCA, Δku80, and *Tg*MCA OE. We observed there was no significant difference (only 1 day) in survival time between mouse in the group infected with 100 tachyzoites of the different strains (Figure 5E). But mice infected with 10 tachyzoites of Δku80 and *Tg*MCA OE showed a significantly delayed time (7–19 days) to death compared to Δ*Tg*MCA (Figure 5F), indicating that Δ*Tg*MCA was more virulent which correlated to the apoptotic function of *Tg*MCA. Furthermore, 25% mouse infected with 10 tachyzoites of *Tg*MCA OE were resistant to the acute inflammatory period and survival. The results above suggest that *Tg*MCA is not involved in parasite growth and replication or function of *Tg*MCA is partially redundant, though a significantly delayed time to death among Δ*Tg*MCA, Δku80, and *Tg*MCA OE at the dose of 10 parasites.

## Discussion

The phenotype of the *Tg*MCA gene complete knockout strain was analyzed in our study. Metacaspases exist in many non-metazoan species, sharing common structural features with caspase. They are able to use the conserved catalytic activity of the cysteine and histidine dyad as a nucleophile to hydrolyze the substrate peptide downstream (McLuskey et al., 2012). A pro-cell death role of metacaspases has been identified in fungi, protozoa, and plants (Meslin et al., 2011; Tsiatsiani et al., 2011). Nyoman et al. showed that caspase-like protease activity was clearly required during miltefosine-induced apoptosis in *T. gondii*, and the caspase inhibitors E64 and Z-VAD-fmk completely abolished drug-induced TUNEL-positive parasites, indicating that caspase-like activity is essential in the apoptotic pathway of *T. gondii* (Nyoman and Lüder, 2013). We have found several caspase-like proteases by a bioinformatics search in ToxoDB, including three ICE family members containing p20 domains and some other cysteine proteases. We found that TGGT1_206490 was the nearest to yeast Yca1, compared to TGGT1_278975 and TGGT_243298. Previous *in vitro* studies of Yca1 have shown that the catalytic residues Cys and His are necessary for hydrolysis (Wong et al., 2012). Therefore, we hypothesize TGGT1_206490 maintains the hydrolysis ability because of the conserved Cys-His catalytic dyad.

We obtained a strain of *T. gondii* tachyzoites lacking the *Tg*MCA gene by gene knockout. First, we analyzed the apoptotic cell death of Δ*Tg*MCA and the parental strains. For apoptosis analysis, Δ*Tg*MCA and Δku80 freshly isolated from the host cell were pre-incubated in extracellular buffer at 37°C for different time. We found that both strains, when freshly isolated (washed the cultures with PBS 3 times and removed the extracellular parasites 3 and harvested the freshly egressed parasites within 30 min), showed no TUNEL-positive signal, and Δ*Tg*MCA and Δku80 showed similar ratio of apoptosis when they were exposed to extracellular buffer for 1 h. Though there was no remarkable difference in the TUNEL labeling assay for 1 h, Δ*Tg*MCA showed greater viability than Δku80 by the method of invasion statistics. Over time, both strains gradually underwent apoptosis during 2–6 h, but the apoptotic ratio of Δ*Tg*MCA remained significantly lower than that of Δku80. In our research, the ratio of apoptotic tachyzoites was different from previous reports (Nyoman and Lüder, 2013) due to different methods of stimulation. In addition, both the *Tg*MCA OE and Δ*Tg*MCA-cm strains were sensitive to the extracellular buffer, which also confirmed the apoptotic function of *Tg*MCA, as the same in some other organisms of which metacaspases over-expressed (Lee et al., 2007; Coll et al., 2010). Based on the process of apoptosis in Δ*Tg*MCA and Δku80, we believe that *Tg*MCA plays a role in regulating tachyzoite apoptosis. Nevertheless, a small quantity of Δ*Tg*MCA were still TUNEL positive, and it is unclear whether other PCD-related proteases might be responsible for the apoptosis of *T. gondii*. Some characterizations of the early stage of apoptosis, such as externalization of phosphatidylserine and loss of mitochondrial membrane potential, have been previously reported, which were not performed in our study. Since caspases are involved in signaling pathways downstream of apoptosis (Carwford and Wells, 2011), the TUNEL assay was an effective and necessary approach in this study. But according to the result of TUNEL assay, the mechanism of the apoptosis of *T. gondii* is still unclear. The alternative experiments such as mitochondrial membrane potential test and the distribution of cytochrome C should be involved to reveal the apoptosis pathway of *T. gondii*, so that the specific *Tg*MCA role would be more strengthened.

The ionic concentration of extracellular buffer was almost the same as the ionic concentration of extracellular fluid. Extracellular *T. gondii* both *in vivo* and *in vitro* undergoes apoptosis (Santos et al., 2011; Nyoman and Lüder, 2013), which indicated that egress tachyzoites which are unable to invade another host cell will undergo active death. DNA fragmentation is among the downstream hallmarks of apoptosis in mammals as well as protozoa (Jiménez-Ruiz et al., 2010; Proto et al., 2013). The cysteine proteases, caspases or metacaspases, are involved in the apoptosis pathway, which may trigger DNA fragmentation directly and indirectly. It is very interesting to observe the different localization of *Tg*MCA in intracellular and extracellular parasites, and the change of localization may reveals to the function of *Tg*MCA, which indicates that it is possible to cleave substrate and trigger DNA fragmentation, ultimately leading to parasite death. However, it is regrettable that we failed to find the downstream substrate peptide that *Tg*MCA target to hydrolyze, as the interaction may have occurred instantaneously. Some studies have indicated that the conserved protein Tudor staphylococcal nuclease (TSN), which participates in activating transcription, is the natural substrate of metacaspase (Sundström et al., 2009). Although TSN exists in *Toxoplasma*, whether it is the target protein of metacaspase and essential for the apoptosis of *Toxoplasma* are an important issue for further study.

Based on the phenotypic assay, Δ*Tg*MCA showed no obvious difference in growth in HFF and Vero cells or virulence *in vivo* compared to Δku80. Therefore, *Tg*MCA is not an obligatory factor for *T. gondii* invasion and proliferation, or redundant proteases are involved. Though *Tg*MCA OE strain showed slower growth *in vitro*, the mechanism hasn't been understood. Up to now, we have generated TGGT1_278975 knockout strain and double genes (TGGT1_278975 and TGGT1_206490) knockout strain (data not shown). We observed that the lack of TGGT1_278975 did not affect the apoptosis of the parasites, but lack of both TGGT1_278975 and TGGT1_206490 has an effect on the proliferation of the parasites significantly (data not shown). We hypothesize the ICE family protein are involved in proliferation of *T. gondii*, but the mechanism has not been clear. Metacaspase studies of other organisms such as yeast (Lee et al., 2010; Hill et al., 2014) and *T. brucei* (Helms et al., 2006) showed multiple functions in protein aggregation and cell proliferation. Whether *Tg*MCA may be responsible for non-cell death functions should be explored in the future. The function of the other two protein have been our focused research recently.

As we all known, virulence assay of mouse infected with dose of 10 tachyzoites are easier to misinterpret as the smaller amount the greater error, and the time taking to count would influence the viability of the parasites. However, we believe that the significant difference in survival time when mouse infected with 10 tachyzoites is due to the much more viability of Δ*Tg*MCA compared to Δku80 and *Tg*MCA OE. The increased invasion number of Δ*Tg*MCA compared to Δku80 when both were pre-incubated in extracellular buffer was also correlated with the apoptotic role of *Tg*MCA in *T. gondii*, as the more viable parasites are, the higher invasion ratio is.

Apoptosis is known in the context of PCD in multicellular individuals, which contributes to the functionality of the organism. Such active cell death has also been observed in unicellular organisms, which seems contrary to evolutionary theory. Despite a lack of evidence regarding the molecular mechanisms of the apoptosis pathway, the benefits of apoptosis as an altruistic behavior have been proposed over the past several years. First, it can control the parasite population to maintain optimal conditions in a host to enable propagation (Bruchhaus et al., 2007). Second, it regulates inflammation of a host by apoptotic parasites induced anti-inflammatory secretions, which seem to be essential for the development of the rest of the population. Van Zandbergen et al. reported that apoptotic *Leishmania* promastigotes could induce TGF-β secretion, thereby enabling the intracellular survival of viable parasites in an altruistic way (van Zandbergen et al., 2006). Another research demonstrated that externalization of phosphatidylserine was necessary for successful *Toxoplasma* infection, thus indicating that PS+ and PS− subpopulations are required to maintain the balance between inflammation and parasite growth (Santos et al., 2011).

In summary, the reduced apoptotic cell death and greater viability of Δ*Tg*MCA indicated that *Tg*MCA performs an important function in *T. gondii* apoptosis. *Tg*MCA was not observed to play a role in propagation from the current result, although multifunction of metacaspases have been confirmed in other species. Our studies demonstrated that *Tg*MCA plays apoptotic role in *T. gondii*.

## Author contributions

QL, JL, and ML conceived and designed the experiments. LM and ML performed the experiments. QL, PH, and ML analyzed the data. QL and JL contributed reagents, materials and analysis tools. QL and ML wrote the paper.

## Funding

This study was supported by National Natural Science Foundation of China (No. 31372424) and the National Key Basic Research Program (973 program) of China (No. 2015CB 150300).

### Conflict of interest statement

The authors declare that the research was conducted in the absence of any commercial or financial relationships that could be construed as a potential conflict of interest.
