# Supplementary material for: The Apoptotic Role of Metacaspase in Toxoplasma gondii
Source: Front Microbiol. 2016 Jan 19;6:1560. doi: 10.3389/fmicb.2015.01560 (PMC4717298; doi:10.3389/fmicb.2015.01560)
Supplement: Supplementary file 1 [file Table1.DOCX]

Table S1. Amino acid similarity of TGGT1_206490, TGGT1_278975, TGGT1_243298 and Yca1 according to DNAman.

|  | TGGT1_206490 | TGGT1_278975 | TGGT1_243298 | Yca1 |
| --- | --- | --- | --- | --- |
| TGGT1_206490 |  | 6.88% | 4.77% | 18.86% |
| TGGT1_278975 |  |  | 6.81% | 5.64% |
| TGGT1_243298 |  |  |  | 2.58% |
