# Supplementary material for: The Apoptotic Role of Metacaspase in Toxoplasma gondii
Source: Front Microbiol. 2016 Jan 19;6:1560. doi: 10.3389/fmicb.2015.01560 (PMC4717298; doi:10.3389/fmicb.2015.01560)
Supplement: Supplementary file 2 [file Table2.DOCX]

Table S2. Primers used for overlapping PCR of *Tg*MCA complete coding sequence

| extron1 | ATGGCGCATGTGAAGCAGCGGTTG |
| --- | --- |
|  | CGACGCCAGACGAGTCAAGTCAAGA |
| extron2 | GTCTCCGCAACTCAGTGTTCCTGTC |
|  | CTTGCTGGATCGACGGACGCG |
| extron3 | CAACAGTCACGGCGTCTGGGCAC |
|  | TCTTCAATTTCTTGTGTGGAGCACTGCG |
| extron4 | AGCCTTGTCGGCGGCCGGCGGCG |
|  | CTGGTGTTCCAACTGATGAACTTGAGTTTCATTTTC |
| P20_1 | AGAAAGCAGTTGATGTCCTCCTGAAGCG |
|  | GTGGACGAATCGTGGAAGCCGTAGACAG |
| P20_2 | CGCAAGGCTCTGCTGGTCGGAATCAACTAC |
|  | GTTGAAGTCGAATTTCTGAGACGACGTCAGC |
| extron10 | GGACTAACTGAGAGCATGGTCTC |
|  | GTCTGTTGAAGTCGAATTTC |
| extron11 | CTTGTCGGATGTGATTCCGAATC |
|  | TCAGTCGTTGAGAAGAGAGCCGA |
