# Supplementary material for: The Apoptotic Role of Metacaspase in Toxoplasma gondii
Source: Front Microbiol. 2016 Jan 19;6:1560. doi: 10.3389/fmicb.2015.01560 (PMC4717298; doi:10.3389/fmicb.2015.01560)
Supplement: Supplementary file 3 [file Table3.DOCX]

Table S3. Primers used for amplifying *Tg*MCA 5’ flanking and 3’ flanking sequences

| 5’ flanking sequence | AGAACCTGCGTCTATTCCAGCCACCTGATC |
| --- | --- |
|  | CTTGCTAAGCAGGCATGTCCCTGCAGTTG |
| 3’ flanking sequence | GGGAGCGATTCAGATTTGTTTTTTTCGAGC |
|  | GGAAAGGAGAAGCAACGAGACAAGAGC |
