# Supplementary material for: The Apoptotic Role of Metacaspase in Toxoplasma gondii
Source: Front Microbiol. 2016 Jan 19;6:1560. doi: 10.3389/fmicb.2015.01560 (PMC4717298; doi:10.3389/fmicb.2015.01560)
Supplement: Supplementary file 4 [file Table4.DOCX]

Table S4. Primers used for identifying Δ*Tg*MCA

| extron2-extron3 | GTCTCCGCAACTCAGTGTTCCTGTC |
| --- | --- |
|  | TCTTCAATTTCTTGTGTGGAGCACTGCG |
| extron10-extron11 | GGACTAACTGAGAGCATGGTCTC |
|  | TCAGTCGTTGAGAAGAGAGCCGA |
| 529 repeat region | CGCTGCAGGGAGGAAGACGAAAGTTG |
|  | CGCTGCAGACACAGTGCATCTGGATT |
