# Supplementary material for: The Apoptotic Role of Metacaspase in Toxoplasma gondii
Source: Front Microbiol. 2016 Jan 19;6:1560. doi: 10.3389/fmicb.2015.01560 (PMC4717298; doi:10.3389/fmicb.2015.01560)
Supplement: Supplementary file 5 [file Table5.DOCX]

Table S5. Primers used for real-time PCR of TGGT1_278975 and TGGT1_243298

| TGGT1_278975 | ACTTCGTCGCGTTTCACTTT |
| --- | --- |
|  | ATGCAGTACGCATTGAGCAG |
| TGGT1_243298 | GACTTGAGAGATAAAGGGATAG |
|  | ACAATCGTTGAAAAGCCG |
