# Supplementary material for: The Apoptotic Role of Metacaspase in Toxoplasma gondii
Source: Front Microbiol. 2016 Jan 19;6:1560. doi: 10.3389/fmicb.2015.01560 (PMC4717298; doi:10.3389/fmicb.2015.01560)
Supplement: Supplementary file 6 [file Image1.PDF]

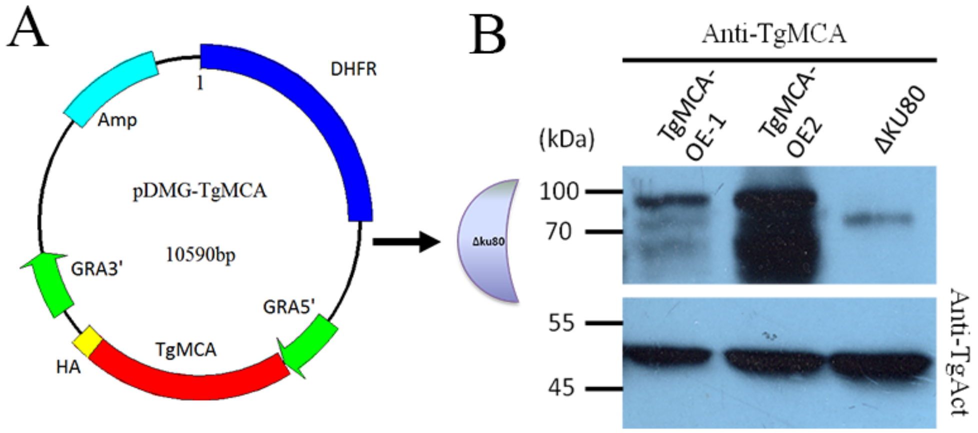

Fig. S1 (A) Schematic of plasmid design about pDMG-*Tg*MCA. A over expression plasmid pDMG-*Tg*MCA was constructed to insert the genome of RHΔku80 to overexpress *Tg*MCA. (B) Western blot analysis of *Tg*MCA over expression using anti-r*Tg*MCA antibody on total extracts from Δku80 and two *Tg*MCA OE clones, and *Tg*Actin was used as control.
