# Supplementary material for: The Apoptotic Role of Metacaspase in Toxoplasma gondii
Source: Front Microbiol. 2016 Jan 19;6:1560. doi: 10.3389/fmicb.2015.01560 (PMC4717298; doi:10.3389/fmicb.2015.01560)
Supplement: Supplementary file 7 [file Image2.PDF]

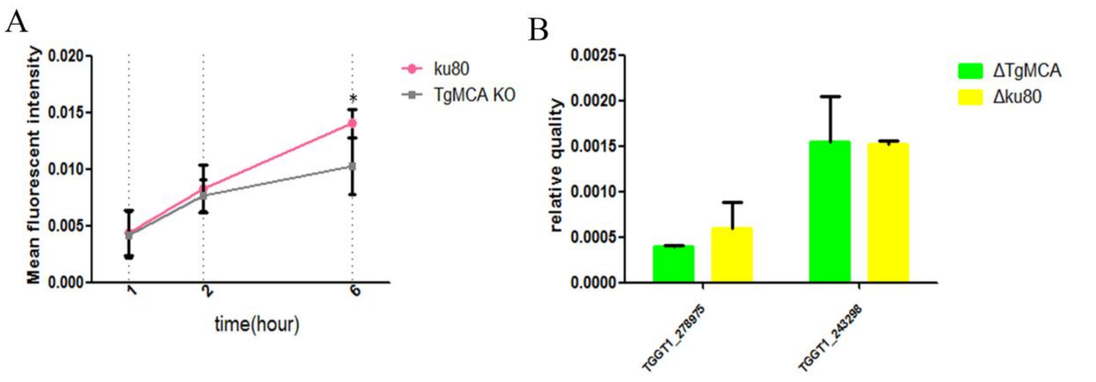

Fig. S2 (A) Mean fluorescence intensity of Δ*Tg*MCA and Δku80 by TUNEL assay. FITC fluorescence intensity of each image was obtained using Leica confocal microscope system at the same fluorescence intensity parameters during image collection. The mean fluorescence was based on the florescence intensity divided by the number of the tachyzoites in each image. The mean fluorescence intensity of Δ*Tg*MCA was significantly reduced compared to Δku80 at 6 hours ($p \leq 0.05$) Asterisks indicate statistically significant results ($p \leq 0.05$) as determined by two way ANOVA with Tukey's post-hoc comparison. Data are mean±SD (error bars) of three independent experiment. (B) The relative transcription level of TGGT1_278975 and TGGT1_243298 of Δ*Tg*MCA and Δku80 by real-time PCR. Asterisks indicated statistically significant results ($p \leq 0.05$, as determined by two-tailed Student's t-test). Data are means ± SD (error bars) of three independent experiments.
